# A 5-year audit of major maxillofacial surgeries at Usmanu Danfodiyo university teaching hospital, Nigeria

**DOI:** 10.1186/s12913-018-3236-1

**Published:** 2018-06-07

**Authors:** Adebayo Aremu Ibikunle, Abdurrazaq Olanrewaju Taiwo, Ramat Oyebunmi Braimah

**Affiliations:** Department of Dental and Maxillofacial Surgery, Usmanu Danfodiyo University Teaching Hospital Sokoto, PMB, Sokoto, 12003 Nigeria

**Keywords:** Audit, Major surgeries, Major maxillofacial surgeries

## Abstract

**Background:**

There is a paucity of data on the pattern of oral and maxillofacial surgeries done in Nigeria. Despite the rising prominence of oral and maxillofacial surgery as a specialty in our immediate environment, no published audit of the surgeries performed exist. This study aims to present the pattern and types of major surgeries done by oral and maxillofacial surgeons in our hospital as well as the indications for such surgeries. It is hoped that the findings will assist in formulating informed policies and improving healthcare delivery.

**Methods:**

A review of hospital records of all patients who had major oral and maxillofacial surgeries at Usmanu Danfodiyo University Teaching Hospital from January, 2013 to August, 2017 was done. Descriptive statistics such biodata, indication for surgery and type of surgery were recorded and analyzed using the IBM SPSS statistics for windows version 20 (Armonk, NY: IBM Corp) software.

**Results:**

One hundred and forty six individuals who underwent 158 major surgeries under general anaesthesia were included. There were 82 males and 64 females, giving a male/female ratio of 1.3: 1. The ages ranged from 3 months to 81 years [median of 33 years]. Tumours and tumour-like lesions were the major indications for surgery [85 (58.2%)]. The most commonly performed surgery was mandibulectomy [31 (19.6%)], followed by Open Reduction and Internal Fixation (ORIF) [29 (18.4%)].

**Conclusion:**

Major oral and maxillofacial surgeries are common in our environment. The frequencies of these surgeries can increase with better healthcare financing and universal availability of health insurance schemes. Efforts aimed at reducing the incidence of tumours should be instituted.

## Background

As healthcare evolves and the demands on the available facilities increase, the need for evaluating existing health systems in order to improve their efficiency becomes more obvious [1]. Such audits play vital roles in research, service delivery improvement and teaching, while also enabling informed distribution of resources [1, 2]. To achieve this, there is often a need to assess the demands being placed on the system, the challenges being experienced and the successes being accomplished. A retrospective review of surgical services provided is one of the ways of ensuring optimal or improved service delivery [3].

The aim of this study is to report the pattern and types of major surgeries done by oral and maxillofacial surgeons in our hospital as well as the indications for such surgeries. Although, reports on audits of oral & maxillofacial surgeries exist in the literature, very few emanate from sub-Saharan Africa. To the best of our knowledge this is the first audit report of major oral and maxillofacial surgeries in our region.

## Methods

This was a retrospective review of the hospital records of all patients who had major surgery at the department of Dental and Maxillofacial Surgery of Usmanu Danfodiyo University Teaching Hospital (UDUTH), between January, 2013 and August, 2017. UDUTH is a tertiary hospital with a 1000-bed facility, which is located in Sokoto state, Northwest Nigeria. It serves as a referral center and caters to the health needs of about 26 million people.

The full-fledged practice of maxillofacial surgery in UDUTH commenced in the year 2011 when a full-time specialist was appointed. However, prior to this time, maxillofacial surgeons were invited from other parts of the country on a locum basis. In the year 2015, two full-time maxillofacial surgeons were employed in addition to the existing one. The dental out-patient clinic attends to an average of 80 patients daily. Averagely, about 4 new cases that need maxillofacial specialist care are seen daily. The mainstay for reconstruction of maxillofacial defects in this center is the use of pedicled flaps, grafts, prostheses or implants. Although expertise in advanced surgeries such as microvascular surgery is available, the practice of this is hampered by limited resources.

The age, sex, indication for surgery, tissue involved, histological diagnosis and type of surgery done were recorded. The analysis was performed using IBM SPSS statistics version 20 [4].

## Results

**Fig. 1:**
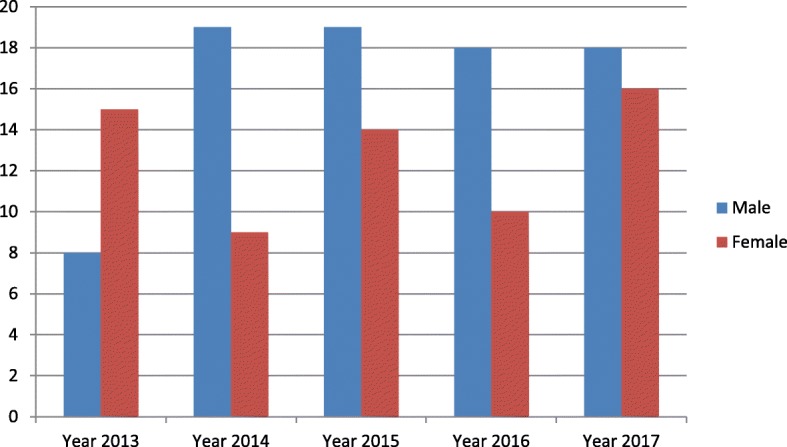
Sex distribution of maxillofacial surgical cases done in UDUTH from year 2013–2017

Records of 146 individuals who underwent 158 major surgeries under general anesthesia were included in the analysis, with an average of 29.2 cases operated upon per annum. The peak number of patients operated was in the year 2017, while the least numbers were observed in the year 2013 (Fig. 1). There were 82 males and 64 females giving a male to female ratio of 1.3: 1. The ages observed ranged from 3 months to 81 years, with a median of 33 years. Majority of the patients were in the fourth decade of life (20.9%, *n* = 27) (Table 1).

**Table 1:** Age and sex distribution of cases done

Age group (years)	Male *N* (%)	Female *N* (%)	Total *N* (%)
0–9	4 (4.9)	6 (9.4)	10 (6.9)
10–19	8 (9.8)	4 (6.3)	12 (8.2)
20–29	13 (15.9)	10 (15.7)	23 (15.8)
30–39	23 (28.1)	13 (20.3)	36 (24.7)
40–49	16 (19.5)	12 (18.9)	28 (19.2)
50–59	8 (9.8)	9 (14.1)	17 (11.6)
60–69	5 (6.1)	7 (11.0)	12 (8.2)
> 70	5 (6.1)	3 (4.7)	8 (5.5)
Total	82 (100%)	64 (100%)	146 (100%)

**Fig. 2:**
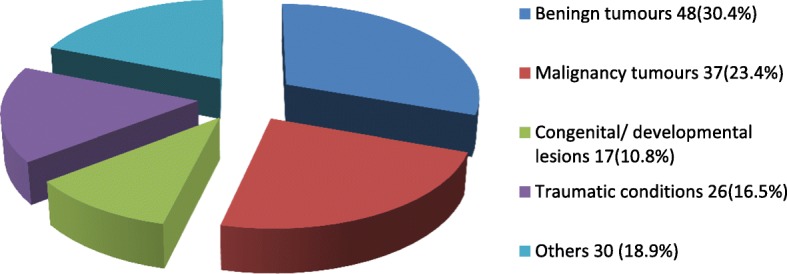
Distribution of the indications for surgery

**Fig. 3:**
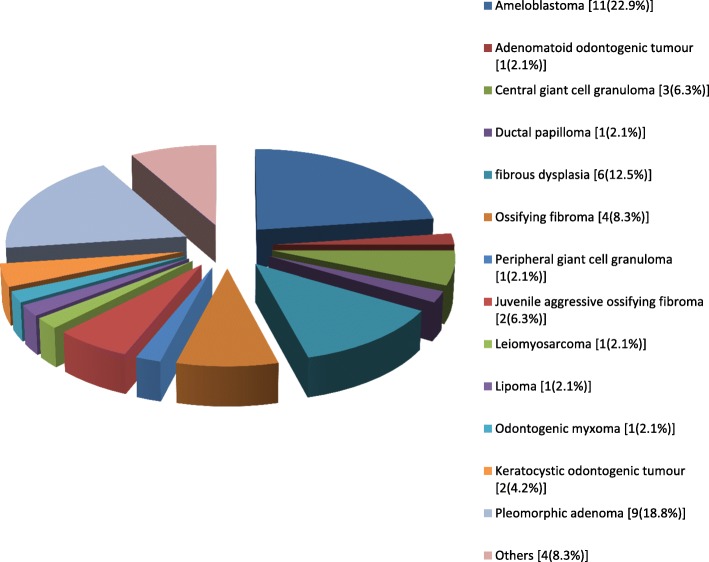
Histological diagnoses of benign tumours/tumour-like lesions seen

**Fig. 4:**
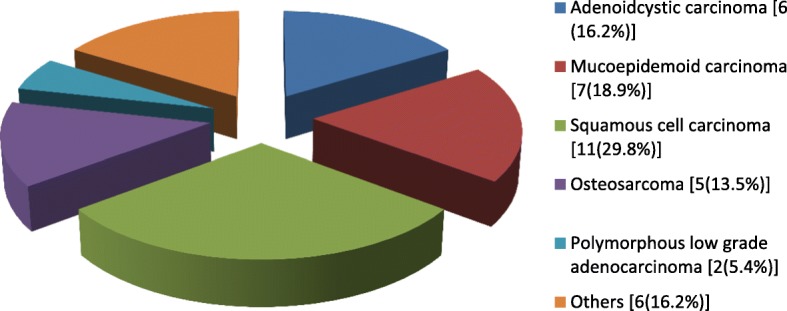
Histological diagnoses of malignant lesions

Tumors and tumor-like lesions were the major indications for surgery, accounting for 85 (58.2%) of the cases (Fig. 2). Benign tumour/tumour-like lesions constituted majority of the tumour/tumour-like lesions seen (56.5%, *n* = 48), while malignancies represented 37 (43.5%). Congenital/developmental conditions (10.8%, *n* = 17), traumatic conditions (16.5%, *n* = 26) and other conditions (18.9%, *n* = 30) accounted for the remaining proportions (Fig. 2). The most frequently histologically diagnosed benign and malignant lesions were ameloblastoma (8.2%, *n* = 12) and squamous cell carcinoma (8.2%, n = 12), respectively (Figs. 3 and 4). Most of the lesions involved both hard and soft tissues at presentation (72.6%, *n* = 61). Hard tissue alone and soft tissue alone were involved in 14 (16.5%) and 10 (11.8%) cases respectively.

**Fig. 5:**
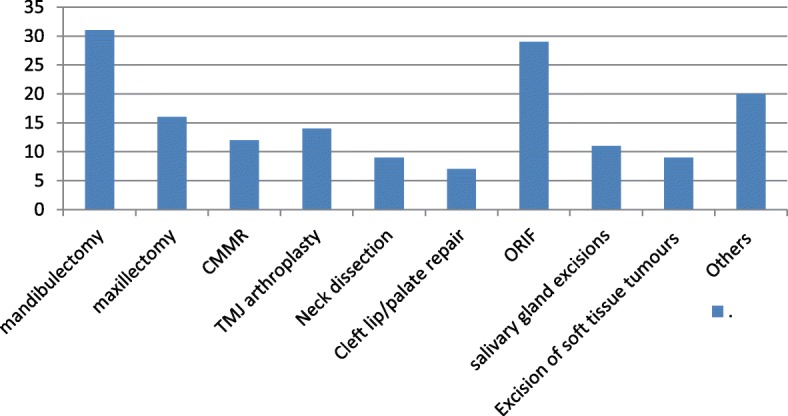
Types of surgeries done

The most commonly performed surgery was mandibulectomy (19.6%, *n* = 31), followed by Open Reduction and Internal Fixation (ORIF) (18.4%, *n* = 29) (Fig. 5). Salivary gland excisions were infrequently done (6.9%, *n* = 11) and they were predominantly parotidectomies (63.6%, *n* = 7) (Fig. 5). Corrective surgeries, composed of cleft lip/palate and temporomandibular joint surgeries, accounted for 18 (12.3%) of the cases, with temporomandibular joint ankylosis constituting majority of them (55.6%, *n* = 10) (Fig. 5).

**Fig. 6:**
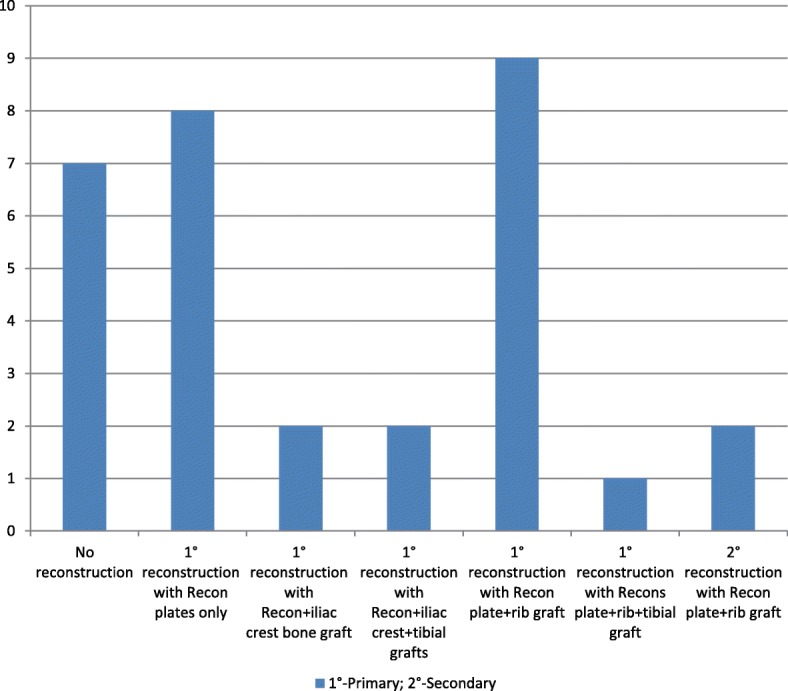
Types of reconstructions done for mandibulectomy defects

Among the cases that had ORIF, wire osteosynthesis was done in 9 (31%) cases, while 2.0 mm mini-plates/reconstruction plates were used in 18 (62.1%) cases and reconstruction plates in 2 cases (6.9%). Immediate reconstruction following tumor ablation was done in 12 (7.6%) patients, while secondary reconstruction was done in 2 (1.3%) (Fig. 6). Mandibulectomy defects were reconstructed in 24 (77.4%) and left unreconstructed in the remaining cases (Fig. 6). Flaps were used in 10 patients with the pectoralis major muscle (20%, *n* = 2), cervical (20%, n = 2) and platysma flaps (20%, n = 2) being the most frequently utilized.

## Discussion

Except for the year 2016, an increase in the number of major surgeries done was noted year after year. This may be a result of increasing awareness of maxillofacial surgery specialty and the services offered among the populace and the medical community [5]. As the awareness increases, there may be a need for expanding the existing infrastructure and manpower. Therefore, policies in this regard may be required.

Majority of the patients treated were males, which is in consonance with several studies in the literature [3, 6, 7]. This may be attributable to the prevalence of paternalistic tendencies in our environment and relative subjugation of females [8, 9]. Additionally, it may be a reflection of the fact that males exhibit a higher prevalence of malignancies than females, worldwide [10, 11]. The frequency of major surgeries performed increased year on year throughout the study period, except for the year 2017.

The age range of the patients is similar to reports in the literature, with most of the patients being in the 4th decade of life [3]. However, some other authors report varying modal decades in their studies, Adebayo et al. and Rehmann et al. reported the 3rd and 1st decades of life as the modal decades in their respective studies [3, 12]. Notably, Rehmann et al. included minor surgical procedures including routine dental extraction in their study, this may have skewed the modal age group recorded [12].

Tumors or tumor like lesions accounted for the majority of the cases done, most of which were benign. This is at variance with some studies in the literature, where traumatic conditions were in the majority [3]. This may be because UDUTH, though, located in an urban area caters for a largely rural population, which is less exposed to the hazards of high-speed traveling [13]. Furthermore, most of the cases of maxillofacial fractures in our environment are treated by closed reduction under local anesthesia, thus potentially reducing the proportion of maxillofacial fracture cases in this study [14].

Malignancies were also frequently seen, the majority of which were Squamous cell carcinomas. This is in agreement with other reports in the literature [15, 16]. The incidence of oropharyngeal squamous cell carcinoma appears to be on an upward trend [17, 18]. This may be attributed to the observed changes in the prevalent biologic drivers of oropharyngeal squamous cell carcinoma, such as the increasing prevalence of infection with high-risk variants of human papilloma virus [18, 19].

The most commonly observed benign tumor was ameloblastoma, which itself is an odontogenic tumor similar to the report of Ajike et al. and Mullapudi et al. [3, 20]. Conversely, Saleh et al., in a study of biopsied oral & maxillofacial lesions reported the most common benign odontogenic tumor to be odontogenic keratocystic tumor [21]. Ameloblastoma has been reported to be the most common odontogenic lesion among differing local populations by various authors [22–24].

Maxillofacial fractures were a prominent indication for surgery in this series. Mandibular fractures were more commonly encountered than any other maxillofacial fracture. This finding is comparable to other reports in the literature [3, 14, 25]. However, it is at variance with some other studies where nasal bone fractures were reported to be the most frequently encountered maxillofacial fracture [26]. Also, Gassner et al. reported maxillary fractures to be the most frequently diagnosed in their study [27]. Mandibular fractures are believed to occur frequently because of its prominence [28, 29].

Maxillofacial fractures often represent a large proportion of the indications for surgery in the oral & maxillofacial specialty in different countries [3, 6]. Maxillofacial fractures were the second most frequent indication for surgery in this study; it may have been a more prominent indication for surgery if all cases of maxillofacial fracture that would have benefitted from ORIF were treated accordingly. Despite the availability of the necessary armamentarium and requisite expertise, patients often opted for closed reduction. Possible reasons for the adoption of closed reduction as reported by various authors range from financial incapacitation on the patients’ part to fear of general anesthesia [14, 25, 30]. Most patients in our climes make out-of-pocket payment for their treatments and this is often challenging [14, 31].

Mandibulectomies were the most frequently performed surgeries. This may be due to the fact that majority of the tumors were ameloblastomas, which are more commonly seen in the mandible than the maxilla [32–34]. A number of Combined Maxillo-mandibular Surgical Resections (CMMSR) was encountered in this study, mostly because of contiguous spread of lesions from one jaw to another. This may be a reflection of the regularity of late presentation among the patients.

Surgical reconstruction of hard tissue ablative defects was done for mandibular defects alone. Rib grafts and reconstruction plates combination was a method of choice because it’s a predominantly compact bone with sufficient rigidity and resistance to infection [35–37]. Moreover, they are suitable for relatively long span defects, which are commonly encountered following tumor ablation in our climes owing to late presentation by the patients [38]. For soft tissue defects that could not be closed primarily, flaps were used. Microvascular surgery is a relatively new frontier in the reconstruction of maxillofacial defects in our climes. It offers considerable advantages over the traditional methods of maxillofacial reconstruction [39, 40]. However, it was not utilized in this study owing to a number of challenges such as limited theatre time, financial incapacitation, inadequate support staff and intensive care unit space constraints.

The few numbers of salivary gland surgeries done may be related to the fact that the specialty of oral and maxillofacial surgery is comparatively new in our hospital and a large percentage of the salivary gland surgeries are referred to other surgical specialties for treatment [41]. With the continued development of the oral and maxillofacial surgery specialty in our locality and the attendant increase in awareness, it is believed that more salivary gland surgeries will be done.

Corrective surgeries accounted for a significant proportion of the surgeries done, with temporomandibular joint ankylosis featuring prominently. This observation is in disagreement with assertions that temporomandibular joint ankylosis is rare [42–44]. The relatively high frequency of Temporomandibular Joint (TMJ) ankylosis observed in this study may be associated with poor health-seeking behavior [45]. Furthermore, cancrum oris, which is often complicated by TMJ ankylosis is still a health concern in our environment [46, 47]. TMJ ankylosis was invariably surgically managed in this study by interpositional arthroplasty. However, the choice of interpositional material varied from the use of the pterygomasseteric muscular sling to the use of the ipsilateral temporalis muscle.

## Conclusion

The field of oral and maxillofacial surgery in our climes is a relatively new one, however, it is evolving and its relevance is growing. A wide range of oral and maxillofacial surgeries, especially those bordering on tumor ablation surgeries, were managed during the study period. With the observed relatively high frequency of tumor ablation surgeries, efforts geared at prevention and postsurgical rehabilitation of patients should be commenced. Additionally, there is a need to improve accessibility to advanced reconstructive procedure, especially with regards to microvascular surgery.

## Abbreviations

CMMSR: Combined Maxillo-mandibular Surgical Resection
ORIF: Open Reduction and Internal Fixation
TMJ: Temporo-mandibular Joint
